# Multiwalled Carbon Nanotube/Graphite Powder Film for Wearable Pressure Sensors with High Sensing Performance

**DOI:** 10.3390/nano12152637

**Published:** 2022-07-30

**Authors:** Shubin Yan, Xiaoyu Zhang, Jilai Liu, Haoqian Xu, Feng Wen, Tingsong Li, Jiamin Cui, Pengwei Liu, Lifang Shen, Yang Cui, Yifeng Ren

**Affiliations:** 1 School of Electrical Engineering, Zhejiang University of Water Resources and Electric Power, Hangzhou 310018, China; zhangxiaoyu9725@163.com (X.Z.); liujl@zjut.edu.cn (J.L.); xuhaoqian0331@163.com (H.X.); lts15296737639@163.com (T.L.); cuijm@zjweu.edu.cn (J.C.); lpw18834800530@163.com (P.L.); shenlf@zjweu.edu.cn (L.S.); cuiy@zjweu.edu.cn (Y.C.); 2 Zhejiang-Belarus Joint Laboratory of Intelligent Equipment and System for Water Conservancy and Hydropower Safety Monitoring, Hangzhou 310018, China; 3 School of Electrical and Control Engineering, North University of China, Taiyuan 030051, China; renyifeng126@126.com; 4 School of Instrument and Electronics, North University of China, Taiyuan 030051, China; nucwenfeng@163.com

**Keywords:** wearable, piezoresistive, multiwalled carbon nanotubes, graphite powder, pressure sensor

## Abstract

With the continuous progress of artificial intelligence and other manufacturing technologies, there is promising potential for wearable piezoresistive sensors in human physiological signal detection and bionic robots. Here, we present a facile solution-mixing process to fabricate a multiwalled carbon nanotube/graphite powder (MWCNT@Gp) film, which has high sensitivity and great linearity and is more oriented to flexible piezoresistive sensors. The sensor consists of two parts: a spinosum microstructure shaped by a sandpaper template and polydimethylsiloxane (PDMS) as the top substrate and interdigital electrodes as the bottom substrate. The experiments we have conducted show that these two parts provide good protection to the MWCNTs@Gp film and improve sensor sensitivity. Additionally, the sensitivity of the optimal ratio of multiwalled carbon nanotubes and graphite powder is analyzed. The 5%MWCNT@5%Gp composites were found to have relatively good conductivity, which is convenient for the fabrication of conductive films of piezoresistive sensors. Finally, we conducted application experiments and found that the flexible piezoresistive sensor can detect minute signals of human motion and different pressure points. This indicates the feasibility of portable sensors in electronic skin and smart devices.

## 1. Introduction

Flexible pressure sensors have drawn increasing attention because of their wide applicability. One application is the monitoring of human motion activities. Usually, pressure sensors are divided into capacitive [1], triboelectric [2], piezoelectric [3], and piezoresistive [4] types. Piezoresistive sensors that transfer external pressure signals into resistance output signals have undergone considerable wearable development due to advantages including real-time response, ultrahigh sensitivity, and large linearity [5]. Given their many advantages, piezoresistive sensors offer a significant platform for a new way to detect real-time and effective human body motion. Overall, however, flexible piezoresistive sensors do not play a vital role in our daily life. One considerable challenge is the trade-off between stability and sensitivity [6]. Generally, increasing sensor sensitivity results in higher measurement accuracy but reduces the measurement range and stability of the sensor to a certain extent. This makes it impossible to measure and identify the object to be measured in a stable way for a long time. To solve this problem, this paper introduces nanomaterials with excellent electrical and mechanical properties as conductive fillers for piezoresistive sensors to maintain stability in sensor measurements. The surface microstructure is constructed to improve the sensitivity of the sensor.

As research in the field of materials science has progressed, various nanomaterials with great mechanical and electrical characteristics have been used in the fabrication of piezoresistive sensors, including carbon black [7,8], graphene [9,10], silver nanowires [11], carbon nanotubes [12], and so on [13]. Among them, multiwalled carbon nanotubes (MWNTs) and graphite powder (Gp) have been demonstrated to be excellent candidates for flexible sensors due to their advantages, such as superior mechanical flexibility, stability, and electrical conductivity [14,15]. However, several researchers in recent years have reported some drawbacks of using single-carbon nanomaterials as conductive fillers for piezoresistive sensors, such as limited mechanical properties and lower sensing performance [16,17]. To eliminate these drawbacks, in this paper, a multiwalled carbon nanotube/graphite powder (MWCNT@Gp) conductive film of a hybrid carbon nanomaterial was fabricated. By studying the literature [18,19,20], the feasibility of carbon hybrid materials as conductive fillers for piezoresistive sensors was determined to further enhance the mechanical and sensing properties of the sensors [21]. 

Due to the high electrical conductivity and low cost of hybrid materials, wearable pressure sensors have been widely developed and studied. Hence, a variety of excellent piezoresistive sensors based on hybrid materials have been evaluated. Yang et al. [22] proposed a piezoresistive sensor that can operate in humid and sweaty environments using a superhydrophobic MXene-coated carboxylated carbon nanotube (C-CNT)/carboxymethyl chitosan (CCS) aerogel with a honeycomb-like porous microstructure as a composite material, which has significant mechanical and electrical characteristics. Zhao et al. [23] proposed a highly sensitive piezoresistive sensor based on carbon nanotube (CNT) sponge composite materials reaching a sensitivity of 4015 ${\mathrm{kPa}}^{-1}$. Xing et al. [24] designed a humidity sensor using a fabric coated with MXene and multiwalled carbon nanotubes as composite materials; these sensors have great application potential for providing critical health information. Zhao et al. [25] proposed a novel flexible tactile sensor using a multilayer ${\mathrm{Ti}}_{2}$C-MXene film as a composite material, and this sensor has a sensitivity of 507 ${\mathrm{kPa}}^{-1}$.

In this study, we design a practical three-layer sensor structure that consists of a top substrate, an intermediate conductive filler, and a bottom substrate. The top substrate is a spinosum surface with a random height distribution, which plays an important role in afferent stimuli for improved pressure perception. This spinosum structure is vital for measuring weak forces and improving sensitivity. The intermediate conductive filler is the MWCNTs@Gp film, which has great stretchability and flexibility and excellent conduction characteristics with external force loading and unloading. We use the most common method of solution mixing to fabricate the MWCNTs@Gp conductive films. The operation of the solution-mixing method is simple and amenable to small sample sizes. Compared with the other common methods of melt blending, the solution-mixing method has a higher efficiency in terms of dispersing MWCNTs and Gp [26] but has restacking and agglomeration issues. To overcome these problems, in this study, MWCNTs dispersed and organic ethanol solvent are added during solution mixing to overcome the van der Waals forces in MWCNTs and ensure proper dispersion and interface bonding between MWCNTs and Gp in PDMS; thus, the sensor can maintain good consistency over long periods of use. In addition, the bottom substrate is composed of a polydimethylsiloxane (PDMS) film and interdigital electrodes, which can protect the conductive film and convert the external pressure signal into an electrical signal. Moreover, various practical applications have been researched, including the detection of large and small human motions. Experiments have shown that the proposed piezoresistive sensor allows tactile sensing of wearable electronics for smart robotics and human motion detection.

## 2. Materials and Methods

### 2.1. Materials

Polydimethylsiloxane (Sylgard 184 silicone elastomer) and curing agent (Sylgard 184 silicone) were purchased from Dow Corning Co., Ltd. (Shanghai, China). Graphite powder (20–50 nm) was purchased from Bocheng Metallurgical Co., Ltd. (Changsha, China). Multiwalled carbon nanotubes were purchased from Tanfeng Tech. Inc. (Suzhou, China). Ethanol absolute ($C_{2}H_{6}O$, analytically pure AR) was purchased from Yatai United Chemical Co., Ltd. (Wuxi, China). Ultra-pure water Level 1 ($H_{2}O$) was purchased from Hewei Medical Technology Co., Ltd. (Guangzhou, China). The interdigitated electrode (10 mm × 10 mm, a line width of 100 µm, a line space of 100 µm) was purchased from Yuxin Electronic Materials Co., Ltd. (Dongguan, China).

### 2.2. Device Fabrication

#### 2.2.1. Fabrication of PDMS Substrates with Abrasive Paper Surface Microstructures

Abrasive paper (no. 150 roughness) was used as the template to produce the spinosum microstructure of the PDMS film; this paper was chosen to ensure excellent sensitivity and conductivity. The PDMS prepolymer was prepared by mixing the base with a curing agent in a weight ratio of 10:1. The PDMS prepolymer was placed in a beaker and stirred with a magnetic stirrer for 15 min. A portion of the PDMS mixture was spin-coated (at 300 rpm) onto the abrasive paper. Then, the PDMS film with a spinosum structure of the abrasive paper was degassed in a vacuum desiccator for 30 min to remove air bubbles. After curing at 70 °C for 2 h, the PDMS film with a spinosum microstructure was peeled off and rinsed twice in absolute ethanol and ultrapure water to remove surface impurities.

#### 2.2.2. Fabrication of the 5%MWCNTs@5%Gp Conductive Film

First, the conductive liquid was produced by adding 1.2 g of multiwalled carbon nanotubes and 1.32 g of graphite powder (5%MWCNTs@5%Gp) into 20 mL ethanol absolute solution. The solution was placed in a beaker and held at room temperature for 10 min with magnetic stirring, which ensured that the material was fully dispersed. Then, 15 mL PDMS was added to the above solution, stirred for 10 min, and evaporated in a blast dryer at a temperature of 70 °C for 1 h to completely evaporate the absolute ethanol. Next, 3 mL PDMS curing agent was added to the solution. This weight ratio was chosen because the filled graphite powder would prevent PDMS silicone gel from reacting with the curing agent, and the weight ratio of 5:1 ensures that the PDMS prepolymer is completely cured. A portion of the mixture was spin-coated (at 500 rpm) onto the glass sheet. Then, the conductive film was degassed in a vacuum desiccator for 30 min to remove air bubbles. After curing at 70 °C for 4 h, the conductive film was peeled off and rinsed twice in absolute ethanol and ultrapure water to remove surface impurities.

### 2.3. Measurement System Setup

The measurement setup consisted of an automatic servo control vertical pressure testing machine (HD-B609B-S) (Haida Instrument Co., Ltd. Xiamen, China) to apply uniaxial compression and release to the sensor surface. The response curve, current, and resistance of the sensor under different pressures are measured by a SourceMeter (Keithley 2420) (Guoxiong Electronic Technology Co., Ltd. Shenzhen, China). The sensor was placed on the stage, and a normal applied force was exerted. Considering the total area of the sensor, the force measurement range was set to 0.0098–0.2156 N, which corresponds to a pressure range of 0.392–8.624 kPa. Each measurement was performed two times and sustained for 5 s to obtain a stable sensor response without being affected by environmental factors. The input voltage was set as 0.1 V during the tests.

## 3. Results

**Figure 1:**
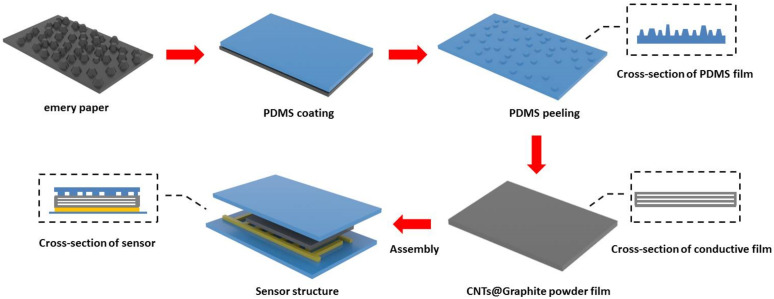
Fabrication process of the three-layer piezoresistive sensor.

Nature often offers inspiration for the manufacture of artificial electronic equipment with bionic structures. As one of the largest organs in the human body, the skin is vital for sensing external stimuli and provides protection. Bioinspired by the function of human skin, a three-layer flexible piezoresistive sensor is proposed. The top substrate of the sensor is PDMS with abrasive paper surface spinosum structures, and the randomly distributed spinosum structure is shown in the cross-sectional schematic The structure has a greater ability to sense minute forces, as the spinosum microstructure generates a local and great stress concentration at the ridge tips. In addition, PDMS films are the most common flexible substrates for wearable piezoresistive sensors due to their excellent elasticity, overwhelming protection, and biocompatibility [27,28]. The fabrication process of the three-layer flexible piezoresistive sensor is shown in Figure 1. PDMS mixed with a curing agent was coated on abrasive paper to obtain a microstructure flexible film. The middle layer is the MWCNTs@Gp film. The distance between Gp and MWCNTs in the conductive film can be seen in the cross-sectional schematic. This conductive film is the crucial film for generating the piezoresistive effect. The bottom substrate is a polyimide (PI)-based commercial interdigital electrode, which has significant mechanical characteristics in terms of stretch ability and stability. The interdigital electrode was laminated onto the conductive film, completing a three-layer structure. Connecting the interdigital electrode with SourceMeter (Keithley 2420) by wire, the electrical response of the device at different pressure levels was measured, resulting in response curve, current, and resistance.

To evaluate the influence of the Gp doping ratio on the MWCNTs@Gp conductive film, five different Gp concentration films, 5%MWCNTs@0%Gp, 5%MWCNTs@1%Gp, 5%MWCNTs@3%Gp, 5%MWCNTs@5%Gp, and 5%MWCNTs@7%Gp, were selected. Among them, 5%MWCNTs@0%Gp is a pure PDMS@MWCNT film, which was taken as the comparison film. We set the MWCNT doping ratio to 5 wt%. The reason is that when the doping ratio of MWCNTs was less than 3 wt%, the MWCNTs were completely wrapped by PDMS, and the number of MWCNTs were not enough to form an effective conductive network in the composite material, which severely inhibited current flow. As the doping ratio of MWCNTs increased, the polymeric distance between adjacent MWCNTs decreased, and a conductive path can be formed. When the doping ratio of MWCNTs reached 5 wt%, a relatively complete conductive grid was formed. When the MWCNTs doping ratio exceeds 5 wt%, the formed conductive grid does not increase further. The composite becomes highly viscous and difficult to flow, leading to difficulties in sensor fabrication and resulting in highly brittle composites [29,30,31,32]. Therefore, to control the only variable and study the influence of Gp′s concentration on the conductive film, the doping ratio of MWCNTs was controlled at 5 wt%.

**Figure 2:**
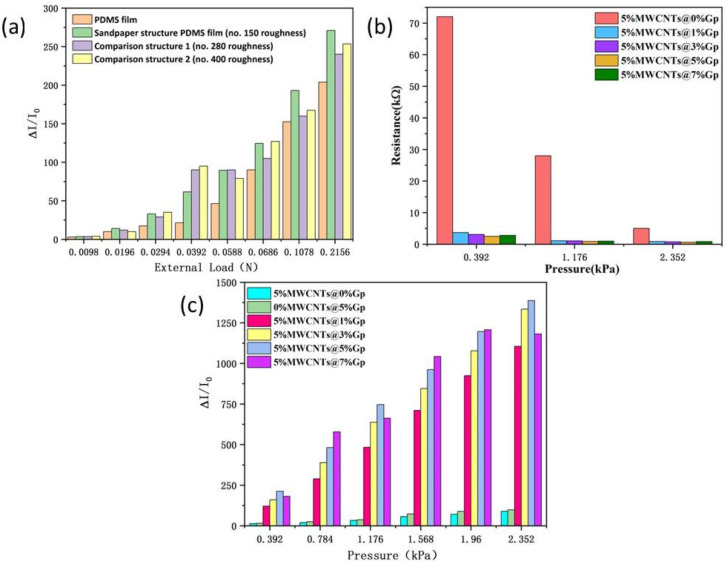
The properties for different doping ratios of Gp. (**a**) Current variation induced by different top substrates under different pressures. (**b**) Resistance variation of different Gp doping ratios under different pressures. (**c**) Current variation induced by different Gp doping ratios under different pressures.

First, the effect of the top substrate on the sensor′s performance was studied. The input voltage was set as 0.1 V during the tests. $I_{0}$ is the current of the sensor under no load, which takes the value of −2 μA. To assess the capacity of the spinosum microstructure of the top substrate to improve conductivity, four structures, with a PDMS film, sandpaper structure PDMS film (no. 150 roughness), comparison structure 1 (no. 280 roughness), comparison structure 2 (no. 400 roughness), were selected. Among them, the 5%MWCNTs@0%Gp film was chosen for the experimental conductive film. Theoretically, the pressure value (kPa) on the sensor can be expressed as $P\left(\mathrm{kPa}\right)=$ Specifically, F(N) denotes the external load (compression force, N) to the sensor, and S($m^{2}$) denotes the effective sensing area. When the external load to the sensor is of a certain value, the spinosum microstructure can effectively reduce the effective sensing area, which results in an increase in the pressure value on the sensor. The current change is shown in Figure 2a. It can be clearly seen from the figure that at the same external load, the sensor with the spinosum microstructure can output a larger current because the spinosum microstructures are conducive to generating a larger pressure value at a small external load, thus improving the sensitivity and conductivity of the piezoresistive sensor. The top substrate based on different sandpaper roughness also has different electrical conductivity, and the sandpaper structure PDMS film (no. 150 roughness) sensor can output a larger and more stable current under the same external load. This can be interpreted as the density distribution of the spinosum microstructure on the surface of sandpaper structure PDMS film (no. 150 roughness), which can better improve the afferent stimulus of pressure perception and avoid the distortion of high roughness comparison structures. Considering the trade-off between sensitivity and linearity, the density distribution of the surface (no. 150 roughness) allows the sensor to steadily output larger current, thus improving the sensitivity and conductivity of the piezoresistive sensor. Subsequently, the influence of Gp concentration on the conductive film was further analyzed by setting the doping ratio of Gp to 0 wt%, 1 wt%, 3 wt%, 5 wt%, and 7 wt%. As shown in Figure 2b, the value of resistance exhibits a pronounced decrease with increasing Gp doping ratio and pressure; this indicates that a large number of Gp and MWCNTs are dispersed in the PDMS to generate a conductive path. The conductivity advantage of the MWCNTs@Gp composites is attributed to the excellent intrinsic electrical conductivity of Gp. When Gp is added to the MWCNTs, the particles in the composites are closer to each other, resulting in more contact and conductive paths. As the Gp content increases, the MWCNT@Gp composite content per unit volume of PDMS increases, while finer and denser conductive networks are constructed. Moreover, due to the synergistic effects of the hybridized network, the network constructed by one-dimensional shaped MWCNTs and two-dimensional shaped Gp had a better elastic modulus [33]. This makes up for the deficiency of single MWCNTs. Please see Table 1 for the resistance values of different Gp doping ratios at different pressures.

Next, the current change in the MWCNTs@Gp film was examined. The input voltage is also set to 0.1 V, and $I_{0}$ is set to −2 μA. Figure 2c illustrates how the current varies with the pressure and the doping ratios of Gp. For films with the same doping ratio, the current increases with pressure. This phenomenon meets the basic requirements of piezoresistive sensor design. Under the same pressure, Figure 2c shows that the current increases dramatically with the doping ratio of Gp. Notably, the current change for 5%MWCNTs@1%Gp is always larger than that of the comparison film. This can be interpreted as the formation of a conductive path to hybrid films becoming simpler with an increasing doping ratio of Gp. Thus, Gp is essential for improving the conductivity of piezoresistive sensors. However, as the ratio of Gp doping increases to 7 wt%, the prepared MWCNTs@Gp film exhibits a poor morphology, and the composite becomes highly viscous and difficult to flow. Excessive Gp content makes it difficult to disperse fully in the composite. This makes the film exhibit a partial agglomeration phenomenon and impedes the batch manufacturing of the conductive film. Figure 2c shows the measured conductivity of the conductive film, in which the conductivity of the 5%MWCNTs@7%Gp film is nonlinear and unstable. This is due to the excessive Gp doping ratio in the composite material, which makes the Gp unable to be easily and fully dispersed in the composite material, so its current growth rate is very small. In summary, the 5%MWCNTs@5%Gp composite material has relatively good conductivity, which is convenient for the fabrication of conductive films for piezoresistive sensors.

**Figure 3:**
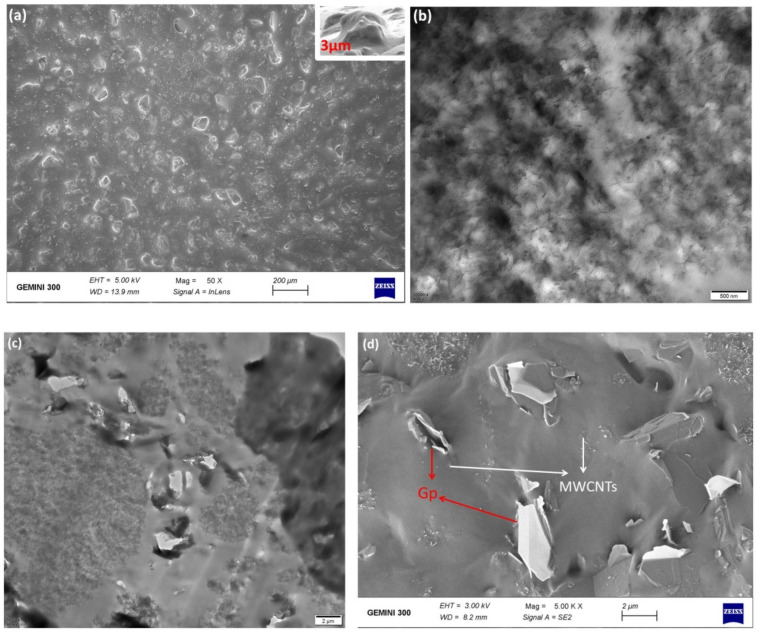
(**a**) SEM image of the top substrate on a scale of 200 μm and 3 μm. (**b**) TEM image of the 5%MWCNTs@5%Gp film on the scale of 500 nm. (**c**) TEM image of the 5%MWCNTs@5%Gp film on a scale of 2 μm. (**d**) SEM image of the 5%MWCNTs@5%Gp film on a scale of 2 μm.

Subsequently, to explore the top substrate and the internal conductivity of the 5%MWCNTs@5%Gp film, we obtained scanning electron microscopy (SEM) images (Figure 3a,d) and transmission electron microscopy (TEM) images (Figure 3b,c). Figure 3a depicts the SEM images of the top substrate. The figure shows that the surface of PDMS film has the randomly distributed spinosum structure. The structure has a greater ability to sense minute forces, as the spinosum microstructure generates a local and great stress concentration at the ridge tips. Both the conductive filler distribution and the interfacial condition between the fillers and matrix are important factors that influence the conductivity performance and stability of the sensor. Hence, it is important to perform morphological studies to better understand conductive composite materials. TEM images of the 5%MWCNTs@5%Gp hybrid material are shown in Figure 3b,c. From the TEM image, the junctions between MWCNTs and Gp are clearly observed, with folds and cracks inside the hybrid films, which may cause the resistance change. Figure 3d shows SEM images of the MWCNTs@Gp@PDMS film on a 2 μm scale. Clearly, the MWCNTs and Gp were well-distributed and embedded in the soft PDMS matrix without aggregation, with MWCNTs distributed randomly and Gp distributed around the network of the MWCNTs, thus interconnecting with each other and improving the mechanical/electrical properties. In addition, some MWCNTs and Gp are not obvious in the SEM image. The reason for this is that the MWCNTs and Gp are parceled inside the PDMS. Due to the good viscosity of PDMS, MWCNTs and Gp can form a more stable conductive network. In other words, the MWCNTs@Gp@PDMS film has a greater ability to protect inner MWCNTs and Gp, as it has high repeatability, stability, and conductivity. 

**Figure 4:**
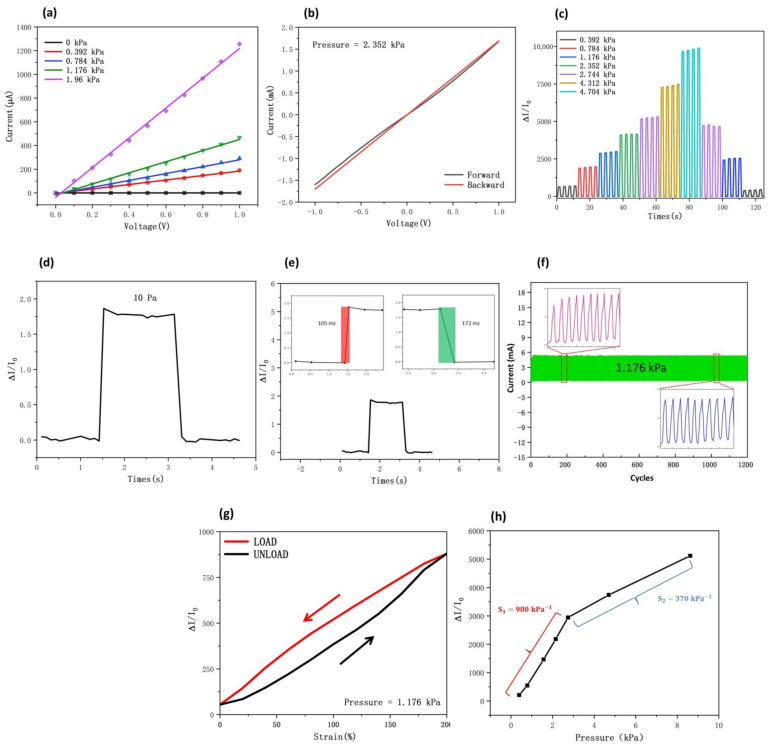

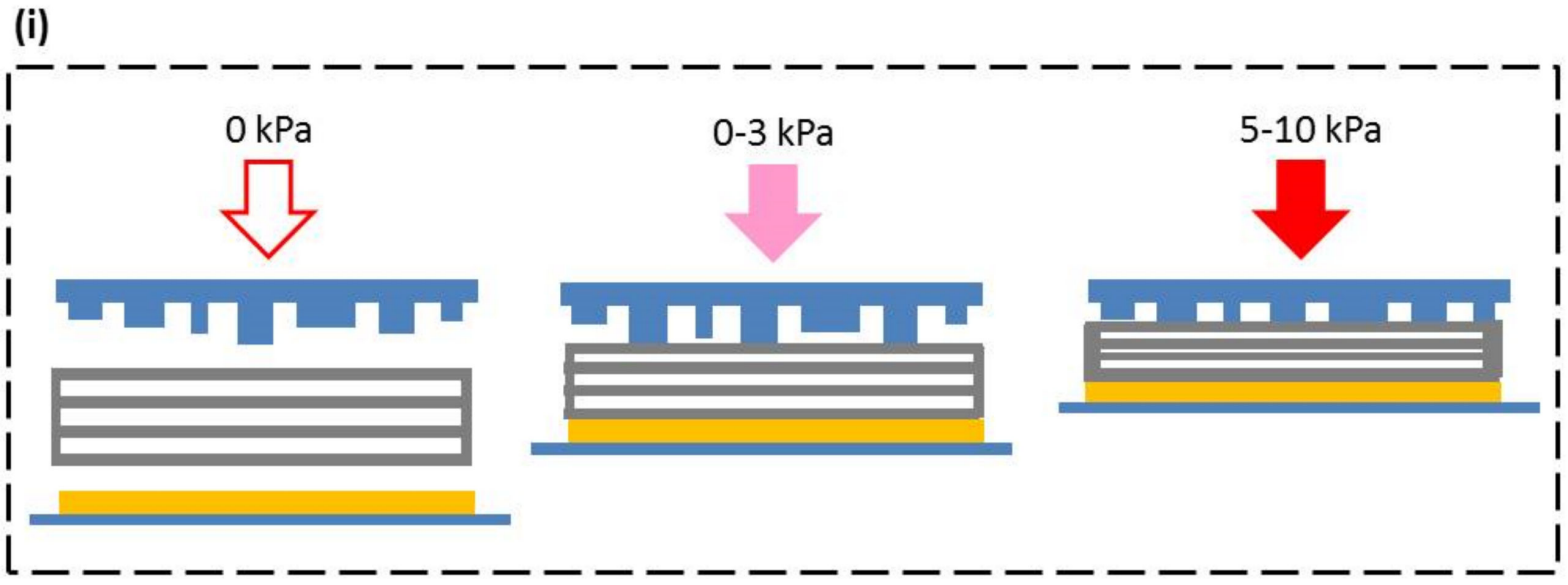
Electromechanical properties of the sensor with 5%MWCNTs@5%Gp conductive film. (**a**) Fitting curves of I-V for different pressures. (**b**) The I-V curves of forward and backward sweeping at a voltage range from −1 to 1 V under a pressure of 2.352 kPa. (**c**) The I-V curves under different pressures. (**d**) The minimum detection of the sensor. (**e**) The rising and releasing time of the sensor. (**f**) Durability of the sensor for 1200 loading/unloading cycles. (**g**) Hysteresis characteristics of the sensor under a pressure of 1.176 kPa. (**h**) Fitting curves of sensitivity for different pressures. (**i**) Schematic diagram of the working principle of the pressure sensor.

To evaluate the electrical response of the designed piezoresistive sensor, we have measured the varying electrical signals under different pressures. The current–voltage (I-V) curves of 5%MWCNTs@5%Gp shown in Figure 4a indicate that when the voltage increases from 0 to 1 V, the conductive film contacts the interdigital electrode and forms an ohmic contact. The slope of the I-V curves is increased, and the curves show strong linear relationships as the external pressure increases. In addition, the I-V curves of forward and backward sweeping at a voltage range from −1 to 1 V were further analyzed. As shown in Figure 4b, under a pressure of 2.352 kPa, the forward curve is almost in accord with the backward curve. This can be interpreted as an excellent ohmic contact between the interdigital electrode and the conductive film. As shown in Figure 4c, the current–voltage curves showed a monotonic rise as the pressure increased, and the current response was steady at the same pressure. When the pressure decreased, the current–voltage curve decreases monotonically, and the current response remains stable at the same pressure. This shows that the piezoresistive sensor is more effective at distinguishing different pressures and keeping stability. The sensor does not suffer mechanical damage due to the pressure changes, and it exhibited consistent, predictable electrical response at different pressures and can thus be applied to the detection of human movement. The minimum detection curve with a pressure of 10 Pa can be clearly seen in Figure 4d, which indicates that this piezoresistive sensor has a great ability to detect minute human motion. Furthermore, the rising time and releasing time were 105 ms and 172 ms, respectively. Figure 4e shows that the proposed piezoresistive sensor can realize real-time body motion testing. The influence of stability on this piezoresistive sensor was further analyzed by loading and unloading pressures for 1200 cycles with a pressure of 1.176 kPa. As shown in Figure 4f, after cyclic testing, the current response remains essentially unchanged, which indicates that the sensor has high repeatability, stability, and durability. The magnified insets clearly show that there is no obvious degeneration during the whole cyclic process; the current signal shows a little attenuation and keeps almost the same current variation after each compression–release cycle, which is important for long-term usage. We further estimated the stretchability and hysteresis characteristics of the piezoresistive sensor. Hysteresis becomes important when the sensor is under dynamic load, for example, which affects the ability of the sensor continue to work stably when it accidentally suffered from very large flexes. Large hysteresis behavior leads to the irreversible sensing performance of sensors upon dynamic loadings. Figure 4g depicts the hysteresis of the sensor at different strains under a pressure of 1.176 kPa. The sensor exhibited consistent, predictable electrical response throughout the load–unload process. The hysteresis (H) of piezoresistive sensor can be expressed as H = (∆$H_{\max}$/$Y_{\max}$) × 100%. Specifically, ∆$H_{\max}$ denotes the maximum difference in the ordinate of the curve at the same pressure during loading and unloading, and $Y_{\max}$ denotes the maximum ordinate in the full range. The hysteresis was 9.82% by calculation, which shows that the sensor still has good reliability under dynamic stress. The curves reached a good basic coincidence during the test. This is because the MWCNTs increases the mechanical properties and reduces the elastic hysteresis. The pores within the whole volume of the 5%MWCNTs@5%Gp also can reduce the volume fraction of the elastomer. As the existence of pores led to reducing the viscoelastic property of the conductive composite materials, a reversible sensor response could be achieved without noticeable hysteresis [34]. It is worthy to mention that the sensor presented gradual and smooth current changes at strain of 0%–200%–0%. This shows that the sensor will not stop working due to accidental bending; it can sense and suffer the scale strain of 0%–200%, and combined with the cyclic testing can show that the sensor has great repeatability, stability, and durability. To evaluate the performance of the proposed piezoresistive sensor, we considered the sensitivity (S), which is defined as $S\;=\left(\Delta I/I_{0}\right)/\Delta P$, where ΔI is the difference in the current before and after applying pressure, ΔP is the pressure change, and $I_{0}$ is the current of the sensor under no load, which means the current before the pressure is applied. Figure 4h illustrates the sensitivity of the designed piezoresistive sensor in two regions; the results show that the sensor exhibits a satisfactory linear fit with the change in pressure, which ensures accurate measurement. After the calculation, the sensitivity is 980 ${\mathrm{kPa}}^{-1}$ in the range of 0–3 kPa, while it has a value of 370 ${\mathrm{kPa}}^{-1}$ in the range of 5–10 kPa. The working principle of the pressure sensor is shown in Figure 4i. When the sensor is subjected to a certain pressure, its conductive composite starts to form a certain contact area with the interdigital electrode. In the 0–3 kPa region, as the abrasive paper surface spinosum structures contact the 5%MWCNTs@5%Gp conductive film, the spinosum structures that first contact the conductive film concentrate the pressure, making the conductive film more compact, and the distance between the GP and MWCNTs atoms rapidly decreases, forming an excellent conductivity path, thus reducing the total resistance of the conductive film and increasing the output current. In the region of 5–10 kPa, most of the spinosum structures are in complete contact with the conductive film, the contact point of the spinosum structure transfers to the periphery, causing the distance between the two atoms in the film to decrease further. However, the pressure from the spinosum structures also increases the contact area between the conductive film and the interdigital electrodes, and the descent rate of the contact resistance decreases, resulting in a decrease in the descent rate of the total resistance of the conductive film. The increase in output current will gradually flatten out. The designed sensor was compared with the pressure sensor mentioned in the first part (Table 2). A comparison of the important parameters of the sensor shows that the 5%MWCNTs@5%Gp conductive film provides good sensitivity, rising time, and releasing time to the piezoresistive sensor. Compared with the excellent piezoresistive sensors in the table, the conductive composite materials of the sensors proposed in this paper are simpler and less expensive to fabricate, do not require too much processing for pristine materials and are suitable for batch production. The use of a spinosum structure and a sandwich structure also greatly increases the sensitivity of the piezoresistive sensor, and the constructed carbon hybrid material conductive network ensures the good consistency and durability of the sensor.

**Figure 5:**
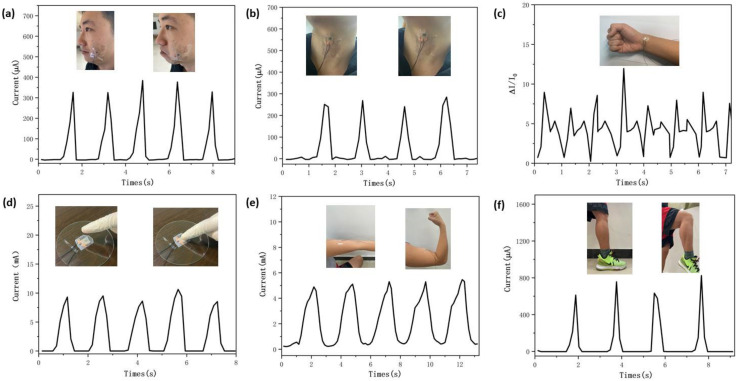
Applications of designed flexible piezoresistive sensors. The I-T curves of (**a**) cheek bulging, (**b**) throat swallowing, (**c**) pulse, (**d**) finger touching, (**e**) arm bending, and (**f**) knee bending.

The designed flexible piezoresistive sensor has ultrahigh sensitivity, great linearity, and excellent stability, which can contribute substantially to the fields of human motion monitoring and artificial intelligence. First, the piezoresistive sensor is attached to the body with scotch tape. This sensor can detect some minute pressure signals, such as cheek bulging, throat swallowing, and pulse, as well as body movements, such as arm and knee bending. Figure 5a illustrates how the current–time (I-T) curves vary with cheek bulging. Each peak of the curve is clearly measured and has a similar shape. Figure 5b,c indicate a different real-time current response of the curves with throat swallowing and pulse, respectively. These results show that this sensor has the potential to distinguish different human physiological activities and has a great ability to detect minute human motion. Additionally, the flexible sensor has a good real-time response and can detect each force and release, as shown in Figure 5d. Similarly, the force of the muscles from the I-T curve can be clearly detected, as shown in Figure 5e. Furthermore, the designed piezoresistive sensor was fixed to the knee to detect bending motion, as shown in Figure 5f. The results show that when the knee bends, the sensor transfers the pressure signal to the current signal. In addition, the current signal remained constant under the same activity. Hence, the designed flexible piezoresistive sensor can potentially be applied to analyze human body movement, such as measuring the force of the muscles during exercise and monitoring the pulse rate.

## 4. Conclusions

This paper presents a practical three-layer piezoresistive sensor structure that consists of a 5%MWCNTs@5%Gp conductive film and a PDMS film based on a sandpaper template. This PDMS film has a spinosum surface with a random height distribution, which plays an important role in the improved sensitivity of the sensor. Five composite material films with different Gp doping ratios were studied, which indicates that the proper doping ratio of Gp in the PDMS/MWCNT composite materials can improve the conductivity of the film. The sensor is designed to achieve a maximum sensitivity of 980 ${\mathrm{kPa}}^{-1}$, which is obviously higher than that of the other piezoresistive sensors mentioned. Furthermore, the sensor exhibits excellent minimum detection, high sensitivities, and fast response and can be applied to human motion detection. This indicates that the proposed sensor can contribute substantially to the fields of electronic skin, artificial intelligence, and other wearable sensing devices.

## Figures and Tables

**Table 1:** Resistance values of different Gp doping ratios at different pressures.

Pressure (kPa)	0%Gp (kΩ)	1%Gp (kΩ)	3%Gp (kΩ)	5%Gp (kΩ)	7%Gp (kΩ)
0.392	72	3.7	3.1	2.5	2.8
1.176	28	1.09	1.03	0.884	0.957
2.352	5	0.871	0.769	0.643	0.829

**Table 2:** Comparisons with other reported piezoresistive sensors.

Reference	Conductive Composite Material	Sensitivity in the Range of 0–5 kPa (KPa^−1^)	Rising and Releasing Time
[22]	F-MXene@C-CNTs/CCS aerogel	3.84	62 ms
[23]	Carbon nanotube sponge	4015	119 ms, 127 ms
[24]	MXene/MWCNT	-	28s under 50% RH, 66 s under 50% RH
[25]	PDMS/Multilayer Ti_2_C-MXene	507	60 ms, 40 ms
This work	PDMS/5%MWCNTs@5%Gp	980	105 ms, 172 ms

## Data Availability

The data presented in this study are available on request from the corresponding author.
